# Identifying volatile metabolite signatures for the diagnosis of bacterial respiratory tract infection using electronic nose technology: A pilot study

**DOI:** 10.1371/journal.pone.0188879

**Published:** 2017-12-18

**Authors:** Joseph M. Lewis, Richard S. Savage, Nicholas J. Beeching, Mike B. J. Beadsworth, Nicholas Feasey, James A. Covington

**Affiliations:** 1 Tropical and Infectious Disease Unit, Royal Liverpool University Hospital, Liverpool, United Kingdom; 2 Wellcome Trust Liverpool Glasgow Centre for Global Health Research, Liverpool, United Kingdom; 3 Department of Clinical Sciences, Liverpool School of Tropical Medicine, Liverpool, United Kingdom; 4 Department of Statistics, University of Warwick, Coventry, United Kingdom; 5 School of Engineering, University of Warwick, Coventry, United Kingdom; National Research Council of Italy, ITALY

## Abstract

**Objectives:**

New point of care diagnostics are urgently needed to reduce the over-prescription of antimicrobials for bacterial respiratory tract infection (RTI). We performed a pilot cross sectional study to assess the feasibility of gas-capillary column ion mobility spectrometer (GC-IMS), for the analysis of volatile organic compounds (VOC) in exhaled breath to diagnose bacterial RTI in hospital inpatients.

**Methods:**

71 patients were prospectively recruited from the Acute Medical Unit of the Royal Liverpool University Hospital between March and May 2016 and classified as confirmed or probable bacterial or viral RTI on the basis of microbiologic, biochemical and radiologic testing. Breath samples were collected at the patient’s bedside directly into the electronic nose device, which recorded a VOC spectrum for each sample. Sparse principal component analysis and sparse logistic regression were used to develop a diagnostic model to classify VOC spectra as being caused by bacterial or non-bacterial RTI.

**Results:**

Summary area under the receiver operator characteristic curve was 0.73 (95% CI 0.61–0.86), summary sensitivity and specificity were 62% (95% CI 41–80%) and 80% (95% CI 64–91%) respectively (p = 0.00147).

**Conclusions:**

GC-IMS analysis of exhaled VOC for the diagnosis of bacterial RTI shows promise in this pilot study and further trials are warranted to assess this technique.

## Introduction

Antimicrobial resistance continues to increase, adversely affecting mortality and morbidity. A major risk factor remains large volume antibiotic prescribing in primary and secondary care[1] and the rise of easily transmissible genetic elements encoding resistance to last-line antimicrobials raises the real possibility of a post-antibiotic era[2]. Rapid and accurate diagnosis would allow reduction in the volume of antimicrobial prescription. Respiratory tract infection remains a major cause of mortality and morbidity worldwide[3] and hence is a driver of antimicrobial prescription. However, in primary care these infections are often viral and self-limiting and antimicrobial prescription is not necessary. For example, it is estimated that of the 40 million antimicrobial prescriptions issued annually for RTI in the United States, 23 million are unnecessary [4]. Thus, there is a pressing need for novel point-of-care (POC) in-vitro diagnostics to reduce the over prescription of antimicrobials in respiratory tract infection (RTI) in primary care.

Analysis of volatile organic compounds (VOC) in exhaled breath is a potential strategy for non-invasive POC diagnosis or exclusion of bacterial infection. To date, analysis of VOCs in human excretions suggests a potential role in the diagnosis of cancers [5–7], inflammatory bowel disease [8], gastric [9] and respiratory infections in chronic obstructive pulmonary disease [10], cystic fibrosis [11], and for the diagnosis of ventilator-associated pneumonia (VAP) [12], amongst other entities. No study has attempted to identify VOC signatures associated with bacterial infection in an unselected population presenting with RTI.

There are a number of available technologies that can be used for the detection of VOCs from clinical samples and/or directly from patients. The gold standard for VOC detection is gas chromatography/mass spectrometry (GCMS), which allows identification of individual VOCs. However, these instruments are large, expensive, cumbersome, usually laboratory based and require specialized staff to operate them and to interpret the data. Thus, the analysis and sample collection from the patient are often separated. Furthermore, for the detection of very low concentration gas phase biomarkers, they require the use of some form of pre-concentration system (typically achieved through the use of absorbent tubes using a material such as Tenax^™^), followed by desorption into the GCMS for analysis.

In an attempt to solve some of these limitations, researchers have applied electronic nose instruments to similar applications. Readers are referred to a recent review for a comprehensive description of electronic nose devices and their potential use in respiratory medicine [13]. In brief, however, these instruments attempt to replicate the biological olfactory system and rely on an array of commercially available gas sensors, with overlapping sensitivities so that they are able to analyze a sample as whole. These instruments can be made smaller, are cheaper than GCMS, use air as the carrier gas and require minimal training to use. Though they have shown promise, many instruments either do not have the required sensitivity nor the repeatability required in a clinical setting (10). A recent development to solve these issues is the gas-capillary column ion mobility spectrometer (GC-IMS). In these instruments, the GC component undertakes some separation of the complex chemical mixture and the IMS detects these separated chemicals with ultra-high sensitivity (down to parts per trillion)[14]. We hypothesized that a GC-IMS instrument could be used to collect exhaled breath samples at the bedside that could distinguish bacterial from viral RTI. We therefore performed a prospective cross sectional proof-of-concept study in hospitalised patients with a diagnosis of RTI in order to assess the feasibility of this technique.

## Materials and methods

### Patient recruitment

The study was carried out in the Royal Liverpool University Hospital, Liverpool, UK; this is a busy 850-bed adult tertiary care facility with around 215,000 attendances to the emergency department in 2015/16. Patients diagnosed with respiratory tract infection by the admitting clinician were prospectively recruited from the Acute Medical Unit (AMU) from 18^th^ March to 17^th^ June 2016 during working hours (Monday-Friday 0900–1700). There were two periods of recruitment; the first ran from 18^th^ March– 20^th^ May, when patients were eligible if they had received a diagnosis of respiratory tract infection from the admitting clinician. Exclusion criteria were: receiving antibiotics between 6 hours and 1 week previously, lacking capacity to consent to be enrolled in the study, too unwell to provide a breath sample or unable to fast for 30 minutes prior to giving a sample, or unable to refrain from drinking water for 15 minutes prior to providing a breath sample. During the second period of enrollment from 21^st^ May– 17^th^ June 2016, the exclusion criterion based on prior antibiotic exposure was withdrawn. In addition, during the second period of recruitment additional patients were included based on virological diagnosis of confirmed viral respiratory tract infection.

Demographic and physiologic parameters were extracted from medical records, along with chest x-ray findings as reported by the radiologist as part of routine care, and the results of blood tests (full blood count, urea and electrolytes and CRP) and any microbiologic investigations performed as part of the patient’s routine care. All investigations were carried out at the discretion of the attending physician, usually aiming to include blood culture, sputum culture (if expectorating sputum) and pharyngeal swab for multiplex RT-PCR for viral pathogens.

#### GC-IMS sample collection

**Fig 1:**
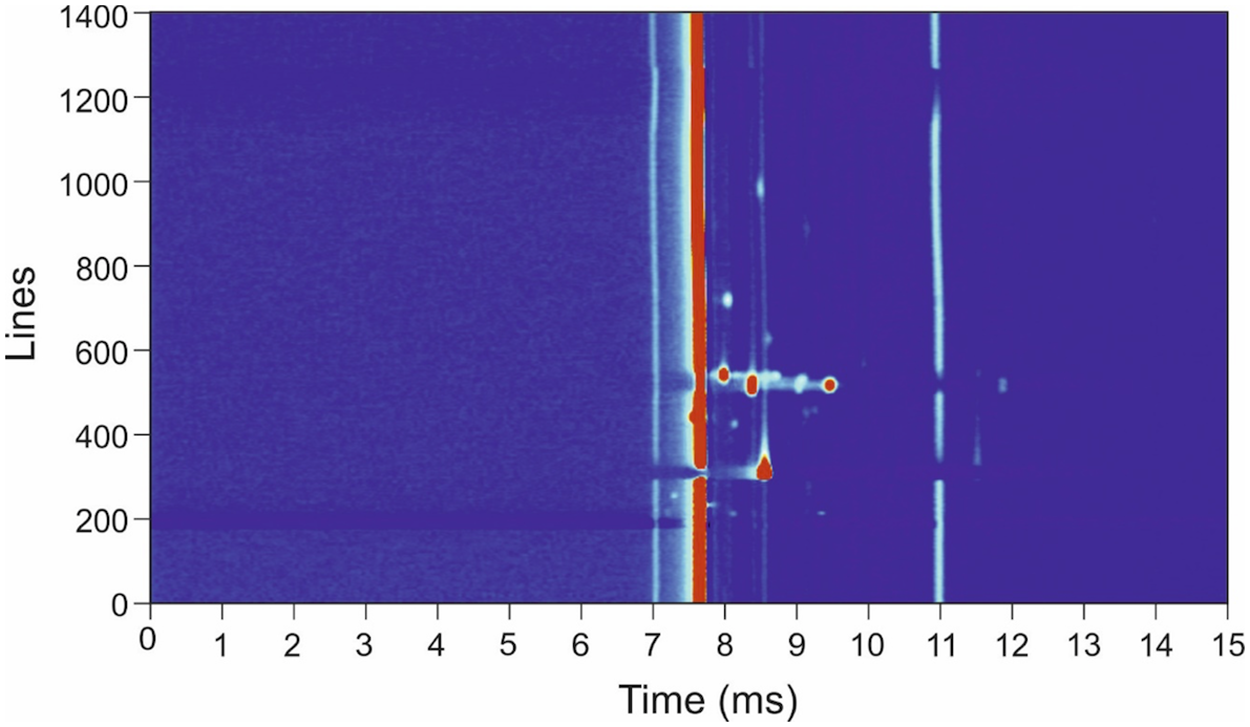
Typical output of the BreathSpec instrument using a breath sample from a confirmed bacterial infected patient.

The gas analysis instrument used was the commercially available Breathspec GC-IMS (IMSPEX, UK), with an attached nitrogen generator (Nitro50L, Lehman, France). This was mounted on a hospital trolley and so could be brought to the patient’s bedside. Patients delivered a breath sample directly into the machine by exhaling into a disposable mouthpiece. The patient was asked to inhale and then exhale a single deep breath. Only the last three seconds of exhaled breath was collected for analysis, ensuring analysis of end-tidal breath only; because of this, the exhaled breath was required to be greater than 3 seconds (typically 5 to 10 seconds). If a patient delivered a breath of less than three seconds (as timed by the device) they were asked to repeat. The results of VOC analyses were stored in the device’s internal memory for later downloading and review. Because of the potential for strong flavours from food and drink to produce erroneous results, all patients were asked to eat nothing and drink only water for 30 minutes prior to providing samples, and to remain completely nil by mouth for 15 minutes prior to providing a sample. Although longer fasting periods have been used by other researchers, in practice within a secondary care setting, these periods of time were the best achievable. An ambient air sample was collected for each breath sample for comparison immediately after collection of the patient breath sample, in the room in which the patient had delivered the sample. This allows the background air contamination to be subtracted from the patient’s sample. The total analysis time for each sample was 10 minutes. Fig 1 shows a typical output from the BreathSpec using a patient sample.

### Case definitions

Patients were classified as confirmed bacterial, viral or probable bacterial or viral on the basis of these investigations, as shown in Table 1. This case definition was chosen to have a high positive predictive value to discriminate between bacterial or viral RTI, rather than to correctly classify all patients, as it was recognised that this was unlikely to be possible with the available tests. If a patient did not meet the confirmed or probable bacterial or viral classification then they were assigned “no classification.”

**Table 1:** Case definitions.

Confirmed bacterial infection	Confirmed viral infection	Probable bacterial infection	Probable viral infection	No classification
Clinical diagnosis of RTI	Clinical diagnosis of RTI	Neither confirmed bacterial or viral infection	Neither confirmed bacterial or viral infection	Neither confirmed bacterial or viral or probable viral or bacterial infection
**AND**	**AND**	**AND**	**AND**	
Typical pathogen cultured from sputum, bronchial washings or blood.	Positive multiplex RT-PCR nose or throat swab or bronchial washings for genotypes of:	Consolidation on chest x-ray	No consolidation on chest x-ray	
**OR**	• Influenza A and B	**AND**	**AND**	
Identification of fastidious “atypical” bacterial organism on multiplex PCR of sputum of bronchial washings:	• Parainfluenza virus 1,2,3,4	CRP > 100 g/dL	CRP < 20g/dL	
	• Respiratory syncytial virus			
• *Mycoplasma pneumoniae*	• Human metapneumovirus			
• *Chlamydia pneumoniae*	• Rhinoviruses			
• *Chlamydia psittaci*	• Enteroviruses			
• *Legionella pneumophila*	• Adenoviruses			
	• Coronaviruses			

### Statistical analysis

All data analysis was undertaken using ‘R’ (R Foundation for Statistical Computing, Vienna, Austria). Summary statistics were calculated to compare the two patient groups: means and standard deviations for normally distributed continuous variables, median and interquartile range for non-normally distributed continuous variables, and proportions for categorical variables. Two sample t-tests were used to compare normally distributed variables between groups, Wilcoxon rank sum test to compare non-normally distributed variables and Fisher’s exact test for proportions. Throughout, a p value of < 0.05 was considered statistically significant. Because of the exploratory nature of this study, no formal power calculations were performed but it was estimated from previous VOC studies that two groups of 20 patients would probably identify any significant differences in VOC spectrum between the two groups.

For the purposes of comparing the GC-IMS data, the patients were split into two groups—“all bacterial RTI”, consisting of all confirmed and probable bacterial RTI and “other”. Data analysis of VOC samples consisted of an unsupervised dimensionality reduction step, followed by a classification step, with 5-fold cross-validation used to assess diagnostic performance.

The GC-IMS data are very high dimensional, so it is convenient to reduce this via a dimensionality reduction (i.e. compression) step. This is appropriate as the information contained within the data are expected to be much lower-dimensional (for example, the number of informative compounds will be much lower than the number of measured values per sample). This is done using sparse principal component analysis, which is known from prior work to give good performance on GC-IMS type data [15]. We used an elbow plot to determine that for these data, 10 sparse principal components (PCs) should be learned from the input data.

The 10-dimensional PCs were then used as input to a 5-fold cross-validation which used sparse logistic regression to predict outcomes. standard pipeline parameter settings were used for the algorithms, to guard against overfitting (i.e. no parameter tuning was performed using the current data set). From this sensitivity, specificity and receiver-operator-curves (with 95% confidence intervals) were calculated.

### Ethical considerations

This study received ethical approval from the NHS South West/Exeter research ethics committee (reference 16/SW/0024). All participants provided written informed consent. Patients without capacity to provide informed consent were not recruited.

## Results

### Participants

**Fig 2:**
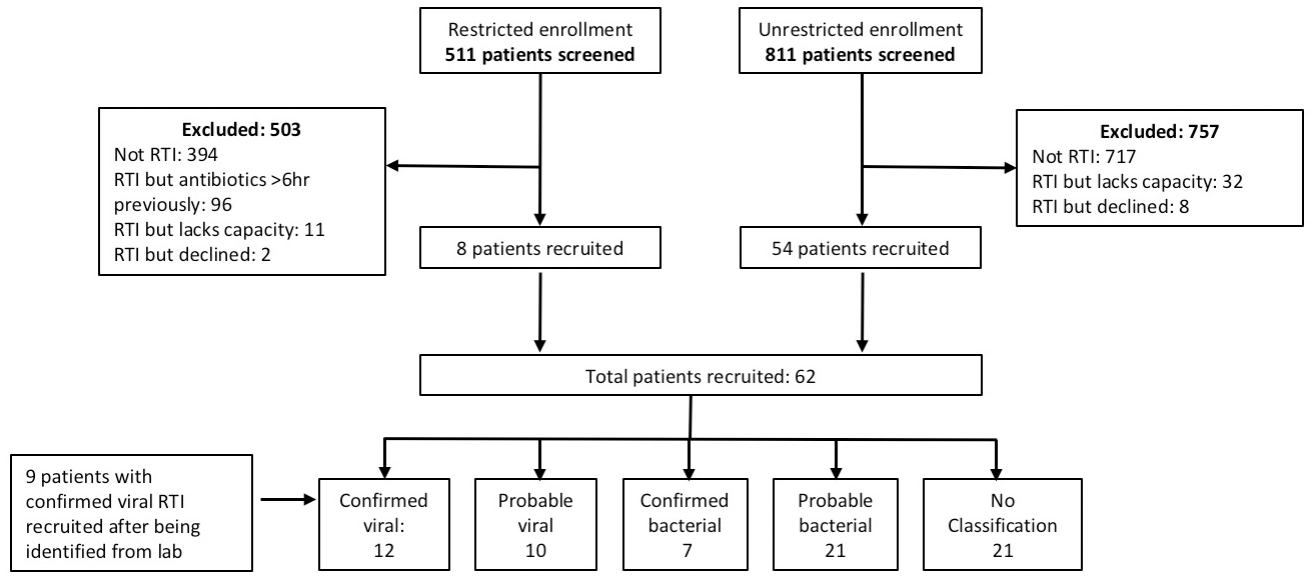
Recruitment flowchart. RTI = respiratory tract infection.

1332 patients were screened in the AMU during the study period and 62 patients recruited; 9 additional patients were recruited via the microbiology laboratory with confirmed viral RTI during the expanded second phase of recruitment. A further 10 patients were approached but declined to be enrolled to the study. Fig 2 shows the flow of patients through the study. Table 2 shows the characteristics of the study participants. There are several statistically significant differences between the groups: an unequal sex distribution; a significantly higher respiratory rate, lower oxygen saturation, higher total white count and neutrophil count, and lower lymphocyte count in the bacterial group; and more benzylpenicillin therapy in the bacterial group.

**Table 2:** Characteristics of study participants grouped by any bacterial infection (confirmed plus probable—Labelled “aggregate bacterial”) and all other participants. Categories for which statistical significance testing for between group differences yields p <0.05 are shown in bold. BP = blood pressure; NEWS = national early warning score; T = temperature; SpO2 = oxygen saturation %; Cr = creatinine; CRP = C reactive protein; CXR = chest x-ray.

	Normal		Aggregate bacterial		Other		p value
	Range						
Total n			28		43		
**Male**		**n (%)**	**21**	***(75%)***	**17**	***(40%)***	**0.004**
Age		median (IQR)	64.5	*(16*.*5)*	67.5	*(23*.*9)*	0.609
Current smoker		n (%)	12	*(43%)*	12	*(28%)*	0.210
Haematologic diagnosis		n (%)	0	*(0%)*	4	*(9%)*	0.148
COPD		n (%)	14	*(50%)*	23	*(53%)*	0.812
Chronic lung disease (inc. COPD)		n (%)	17	*(61%)*	26	*(60%)*	1.000
**Received benzylpenicillin**		**n (%)**	**14**	***(50%)***	**10**	***(23%)***	**0.024**
Received clarithromycin		n (%)	15	*(54%)*	17	*(40%)*	0.330
Received Doxycycline		n (%)	6	*(21%)*	14	*(33%)*	0.420
Received amoxicillin		n (%)	5	*(11%)*	6	*(14%)*	0.742
Received Piperacillin/tazobactam		n (%)	3	*(11%)*	11	*(26%)*	0.143
Received teicoplanin		n (%)	4	*(14%)*	1	*(2%)*	0.075
Received gentamycin		n (%)	0	*(0%)*	4	*(9%)*	0.148
Received oseltamivir		n (%)	0	*(0%)*	1	*(2%)*	1.000
Number of antibiotics		median (IQR)	2	*(1)*	2	*(1)*	0.007
Received steroids		n (%)	11	*(39%)*	21	*(49%)*	0.472
Received nebulisers		n (%)	16	*(57%)*	23	*(53%)*	0.811
Systolic BP (mmHg)		mean (SD)	126.4	*(21*.*0)*	126.7	*(17*.*0)*	0.952
Diastolic BP (mmHg)		mean (SD)	73.8	*(11*.*1)*	71.9	*(11*.*9)*	0.493
**Resp. rate (/min)**		**mean (SD)**	**20.4**	***(3*.*0)***	**19.0**	***(2*.*2)***	**0.045**
T/C		mean (SD)	36.7	*(0*.*3)*	36.6	*(0*.*2)*	0.369
Heart rate(/min)		mean (SD)	92.4	*(10*.*2)*	86.9	*(19*.*4)*	0.123
**SpO2**		**mean (SD)**	**0.92**	***(0*.*03)***	**0.94**	***(0*.*03)***	**0.007**
Received supplementary O2		n (%)	14	*(50%)*	21	*(49%)*	1.000
Lactate (mmol/L)	< 2	mean (SD)	2.0	*(1*.*0)*	1.7	*(0*.*5)*	0.150
Hb (g/L)	115–165 (female)	mean (SD)	123.3	*(26*.*7)*	125.2	*(19*.*2)*	0.752
	130–180 (male)						
**WCC (x10**^**9**^**/L)**	**3.6–11.0**	**mean (SD)**	**15.0**	***(5*.*6)***	**11.3**	***(5*.*0)***	**0.010**
**Neutrophils(x10**^**9**^**/L)**	**1.8–7.5**	**mean (SD)**	**12.7**	***(6*.*0)***	**8.7**	***(4*.*8)***	**0.007**
**Lymphocytes (x10**^**9**^**/L)**	**1.0–4.0**	**mean (SD)**	**1.1**	***(0*.*4)***	**1.5**	***(1*.*1)***	**0.050**
Platelets (x10^9^/L)	150–450	mean (SD)	238.6	*(109*.*4)*	259.7	*(111*.*4)*	0.447
Sodium (mmol/L)	135–145	mean (SD)	134.7	*(7*.*2)*	137.6	*(3*.*4)*	0.053
Potassium (mmol/L)	3.4–5.0	mean (SD)	4.2	*(0*.*5)*	4.1	*(0*.*4)*	0.613
Urea (mmol/L)	2.5–7.0	mean (SD)	6.0	*(6*.*0)*	6.2	*(3*.*7)*	0.913
Cr (mmol/L)	49–90 (female)						
	64–104 (male)	mean (SD)	74.6	*(23*.*5)*	88.7	*(67*.*3)*	0.220
**CRP (g/dL)**	**<5**	**mean (SD)**	**164.8**	***(124*.*5)***	**56.1**	***(66*.*0)***	**<0.001**
**Consolidation on CXR**		**n (%)**	**27**	***(96%)***	**8**	***(19%)***	**<0.001**
Hours since antibiotics		mean (SD)	28.3	*(22*.*0)*	34.6	*(52*.*1)*	0.486
**Any micro samples sent**		**n (%)**	**23**	***(82%)***	**25**	***(58%)***	**0.041**

### Microbiologic investigations and results

A total of 48 of 71 patients (68%) had at least one microbiologic sample sent. The details of the samples sent and pathogens identified are shown in Table 3.

**Table 3:** Microbiologic samples sent.

Microbiologic sample	Number (%) of patients for whom sample was sent	Number (%) of patients with sample positive for pathogen	Pathogens identified (n)
Blood Culture	34/71 (48%)	1/34 (3%)	*Staph*. *aureus* (1)
Sputum culture	18/71 (25%)	6/18 (33%)	*Haemophilus influenzae (4)* *Streptococcus pneumoniae (1)* *Klebsiella pnemoniae (1)*
Nose/ throat swab for viral multiplex RT-PCR	15/71 (21%)	12/15 (80%)	Influenza B (4)* Adenovirus (3)* Rhinovirus (3) Coronavirus (2) Human metapneumovirus (1)
Urine pneumococcal/ legionella antigen	3/71 (4%)	0 (0%)	None
Pleural fluid culture	2/71 (3%)	0 (0%)	None

* = one sample from one patient was positive for both Influenza B and adenovirus.

### VOC analysis

**Fig 3:**
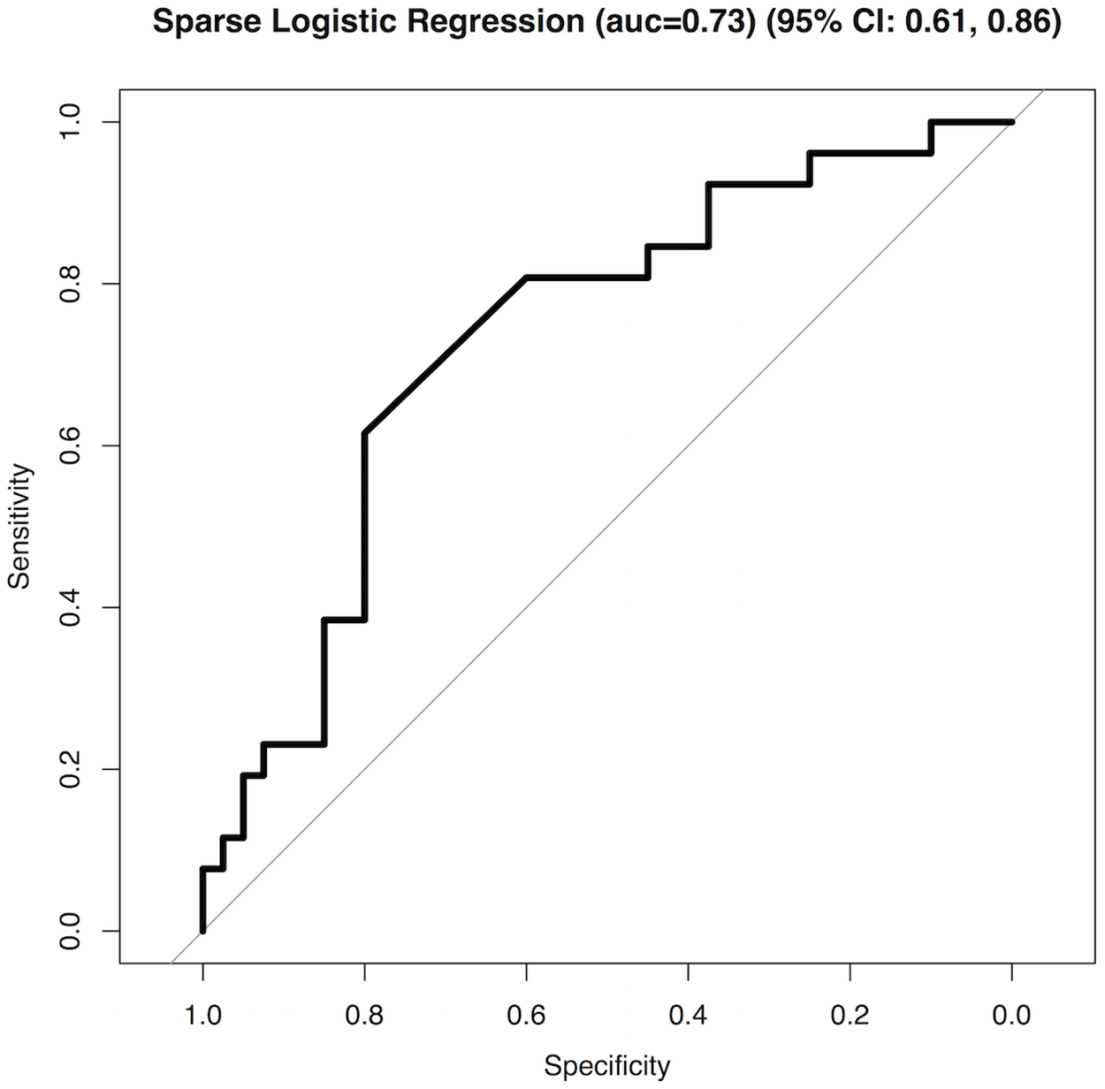
Receiver operator characteristic curve for final diagnostic model. AUC = area under the curve.

The final diagnostic model produced an area under the receiver operator characteristic curve (AUC- ROC) of 0.73 (95% CI 0.61–0.86) when discriminating between “all bacterial” and “all other” samples. The summary test characteristics were; sensitivity of 62% (95% CI 41–80%) and specificity of 80% (95% CI 64–91%) (Fig 3).

## Conclusions

This study demonstrates, for the first time, the feasibility of VOC analysis using a portable GC-IMS instrument on exhaled breath samples, at the patient’s bedside, to identify bacterial RTI in unselected medical inpatients with an admitting diagnosis of RTI. The accuracy in this cohort was moderate: AUC 0.73 (95% CI 0.61–0.86). The testing procedure was well tolerated, and all patients who agreed to provide breath samples were able to do so, despite being hospitalized and around 50% of patients receiving supplemental oxygen. The vast majority (71/81 [88%]) of eligible patients who were approached agreed to participate.

There are several limitations to this study, which mean that the results should be interpreted with caution. Most of these are either due to the small sample size or the exploratory nature of this study. Firstly, the diagnostic algorithm has not been validated in an external cohort. Secondly, rates of microbiologic testing that were performed on the cohort were low: 25% of patients had sputum culture sent and 21% had throat swab sent for viral multiplex PCR). As a result, there were few patients with confirmed bacterial and viral infections and the majority of patients were classified as probable bacterial or viral RTI on the basis of proxy radiographic and biochemical tests (chest radiography and CRP), rather than microbiologic tests. Although there is some evidence that a CRP of greater than 100mg/L is associated with bacterial RTI and a CRP of less than 20mg/L with viral RTI[16], CRP is an imperfect test for this purpose. As CRP is a marker of systemic inflammation it is also possible that defining our groups in the way we have done will split them by disease severity, rather than by aetiology. There is some evidence that this is the case, with the “all bacterial RTI” group having a significantly lower oxygen saturation and higher respiratory rate, both markers of severity of disease. In this situation, any differing VOC signature could be related to severity specific host responses to disease, rather than the aetiologic agent.

The low rates of microbiologic testing in our cohort also raises the possibility of selection bias, in that the patients who were chosen for sputum culture or viral multiplex PCR by their attending clinician may be different in some way from those who did not undergo testing. This is made more problematic by the fact that the low numbers of positive viral PCR results forced us to recruit patients identified in the laboratory to have positive results; in fact, the majority of the patients in the “confirmed viral RTI” group were recruited in this way. This is another potential source of selection bias that could introduce a difference between the “all bacterial RTI” and “other RTI” groups. The problem of low rates of identification of a causative organism is one that is common in both LRTI aetiology studies and clinical care[17].

Thirdly, because of the exploratory nature of this study small numbers precluded an analysis that takes into account diseases that are known to affect the VOC profile (diabetes mellitus, COPD, etc.). Fourthly, a decision was made not to use a wash-in (where the participant breathes clean or scrubbed air prior to exhaling into the device), which in principle could have resulted in ambient air VOCs being erroneously attributed to the patient. This decision was made because it may have been uncomfortable for our patient population of interest; that is, patients hospitalized with respiratory tract infection. To mitigate against misattribution of ambient air samples, a sample of room air was collected for each patient. In the event, the background air variations were small. In addition, as the unit uses a GC pre-separation it is likely that interfering molecules will be detected separately to any biomarkers, so a wash in is not critical in this situation.

Finally, the sensitivity and specificity of the technique are at best modest; it may be that the heterogeneity of the study population, in addition to the factors above, has contributed to this. Notwithstanding these limitations, this pilot study has demonstrated the feasibility of beside point VOC in a hospitalized, unselected RTI population. Further clinical studies are needed to validate this technique in much larger patient sets, using comprehensive diagnostic microbiology and robust case definitions agreed by an expert panel. This will enable a distinction to be made between patients with different underlying conditions such as emphysema or diabetes.
